# Design and Tracking Control Experimental Study of a Hybrid Reluctance-Actuated Fast Steering Mirror with an Integrated Sensing Unit

**DOI:** 10.3390/s25030910

**Published:** 2025-02-03

**Authors:** Jian Zhou, Yudong Fan, Liang Li, Feng Zhang, Bo Feng, Minglong Xu

**Affiliations:** 1 State Key Laboratory for Strength and Vibration of Mechanical Structures, School of Aerospace Engineering, Xi’an Jiaotong University, Xi’an 710049, China; zhoujian163@xjtu.edu.cn (J.Z.); liliang870130@stu.xjtu.edu.cn (L.L.); zhang_feng@xjtu.edu.cn (F.Z.); bofeng@xjtu.edu.cn (B.F.); 2 Xi’an Aerospace Propulsion Institute, Xi’an 710100, China; fyd0126@outlook.com

**Keywords:** fast steering mirror, reluctance actuator, strain sensor, tracking control, adaptive inverse control

## Abstract

This study proposes the design of a hybrid reluctance-actuated fast steering mirror (HRAFSM) using Maxwell’s electromagnetic normal stress principle. Strain gauges were attached to the flexible supports as sensors for measuring the rotation angles. According to Maxwell’s stress tensor theory and the theory of vibration mechanics, we obtained the dynamic equation of the HRAFSM in the uniaxial direction to investigate the relationship between the input current and the output angle of the entire system. Further, we propose a control algorithm combining proportional-integral-derivative (PID) and adaptive inverse control (AIC) to achieve high-precision control. We established an experimental system for testing and validation of the control method. The experimental results showed that the designed HRAFSM can achieve the expected rotation angle of ±1.5 mrad, and revealed a linear relationship between the rotation angle of the two axes and their corresponding strain voltages. The effectiveness of the designed controller was verified, and the amplitude tracking errors of the x- and y-axes were 0.1% and 0.14%, respectively.

## 1. Introduction

In order to achieve wide range tracking and arcsecond-level aiming of maneuvering targets, it is necessary to continuously improve the performance of the beam precision pointing device. A fast steering mirror (FSM) is a device with a controllable payload for rapid movement with single or multiple degrees of freedom, which allows quick changes in the beam propagation direction. FSMs are widely used in optical systems, such as laser communication systems [1] and high-resolution reconnaissance satellites [2]. It can perform stable beam scanning, optical path modulation, and image offset compensation in the optical system, achieving high-precision target tracking and capture. An FSM mainly consists of the reflector, drivers, flexible support structure, angle measurement sensors, the base, and the control system. For a two-axis rotating FSM, its basic working principle is that the angle measurement sensor measures the rotation angle and feeds it back to the controller, and then the controller integrated with the control algorithm can closed-loop control the drivers to achieve the target angle by pushing and pulling.

At present, FSMs are primarily driven by voice coil motors [3,4,5] and piezoelectric ceramics [6,7,8]. Voice actuators follow the Lorentz force principle, which can achieve large displacements at low voltages. However, this mechanism faces certain limitations, such as limited output force, excessive heat generation, and a small control bandwidth. The FSMs designed with the voice coil motors generally achieve a rotation range of more than ±1°, with a control bandwidth below 500 Hz. In comparison, the piezoelectric actuators exhibit a higher output force and bandwidth, but a smaller output displacement. Moreover, they require a large actuation voltage. The FSMs designed with the piezoelectric actuators generally achieve a rotation range of less than ±2 mrad, with a control bandwidth over 1.0 kHz. An ideal actuator should combine the advantages of both voice coil motors and piezoelectric ceramics to achieve better output force, displacement, and bandwidth at low voltages. The hybrid reluctance drive using Maxwell’s electromagnetic normal stress principle can achieve this goal [9]. Kluk [10] proposed designs for a new class of fast steering mirrors using the hybrid reluctance drive, named advanced fast steering mirrors (AFSMs). This AFSM has a travel of ±3.5 mrad. Csencsics [11] developed a HRAFSM system using eddy current sensors, achieving a closed-loop control bandwidth of 1.0 kHz. They further designed a compact and highly integrated FSM, with eddy current sensors built into the structure [12]. A closed-loop bandwidth of 1.5 kHz was achieved within a small range of Φ32 × 30 mm^2^. Zhang [13] provided a detailed theory and simulation design method for an HRAFSM. However, the corresponding products are yet to be manufactured and tested. Overall, AFSMs can easily achieve a rotation range of over ±3 mrad, and due to the high output force of the hybrid reluctance drive, AFSMs can be designed with a higher fundamental frequency, thus generally achieving a control bandwidth of over 500 Hz.

Appropriate sensor selection is essential to achieve high-precision FSM control. Strain gauges have been employed as sensors in piezoelectric ceramic-driven FSMs [14], whereas non-contact displacement sensors, such as eddy current sensors [15], inductive position sensors [16,17], and four-quadrant detectors [1], have been applied to electromagnetic FSMs. Eddy current sensors are the main sensing method of HRAFSMs [10,11,12]. Although these sensors exhibit high enough accuracy and resolution, they require additional sensor support design [15], which increases the structure’s size and complexity. Applying strain sensing to the HRAFSM can help achieve a compact design.

In order to achieve the closed-loop control of the FSM, a control algorithm design is essential. Proportional-integral-derivative (PID) and its improved control methods are the most widely used [7,11,15]. Some more advanced control methods have also been applied to the control of FSMs, such as model reference adaptive control [18], robust finite-time adaptive control [19], and robust current control based on a disturbance observer framework [20]. These control methods have been experimentally validated for their effectiveness. However, for research on the control algorithm of the HRAFSM, only the PID method was used. In order to improve the control accuracy and make it suitable for engineering applications, a control algorithm combining PID and feedforward signals (adaptive inverse control, AIC) was adopted.

On the basis of the analysis above, in this study, we propose an HRAFSM with integrated strain sensing, with a control algorithm combining PID and AIC, i.e., PID + AIC, to achieve a compact HRAFSM design and high-precision control.

## 2. Structural Design and Operating Principle

**Figure 1:**
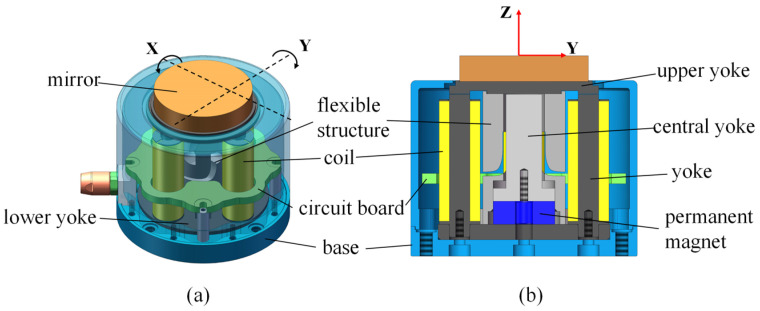
(**a**) Three-dimensional and (**b**) cross-sectional structure of the HRAFSM.

The proposed HRAFSM mainly consisted of a mirror, an upper yoke, a flexible structure, a central yoke, a permanent magnet, two sets of orthogonal electromagnetic drive units, a lower yoke, a circuit board, and a base (Figure 1). The mirror was fixed on the upper yoke, which was, in turn, fixedly connected to the flexible structure with bolts. The lower yoke, with a groove at the center for fixing the permanent magnet, was placed between the flexure hinge connected to the base via a through-hole at the bolt’s passing position. The central yoke was located above the permanent magnet, fixed to the base with bolts. This setup allowed an air gap between the central and upper yokes. Four orthogonal through-holes were drilled on the outer ring of the lower yoke for installing the electromagnetic drive unit. The circuit board used for processing strain signals was integrated into the structure.

**Figure 2:**
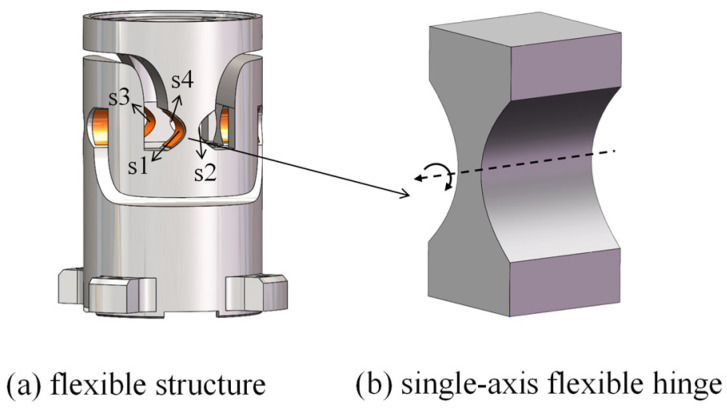
Diagrammatic representation of the flexible HRAFSM structure.

The flexible structure consisted of four single-axis parabolic flexure hinges, as shown in Figure 2b. These flexure hinges were first connected in parallel and then in series to form a decoupled guiding mechanism with two degrees of freedom (DoF). To obtain the integrated design of the driving and sensing mechanism, two strain gauges were attached to each of the four flexure hinges as sensors. We designed two sets of Wheatstone bridge strain sensors to measure the two rotational DoF. The location of one set of strain gauges is shown in Figure 2a. Strain gauges s1 and s2 were attached to both sides of one flexure hinge, and s3 and s4 were attached to both sides of another flexure hinge.

**Figure 3:**
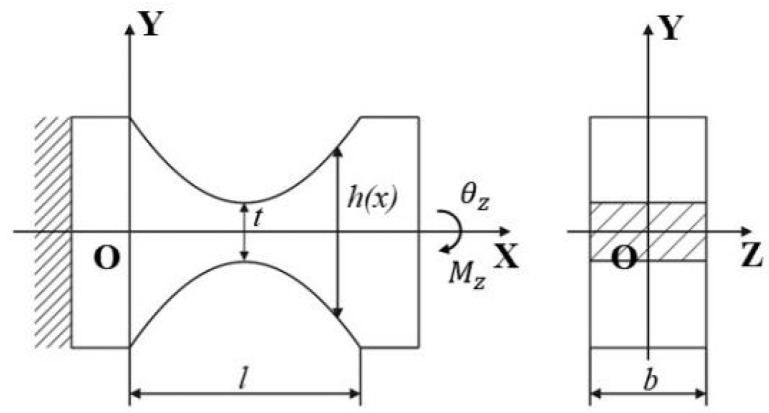
A single-axis parabolic flexible hinge.

As a guiding component for the mechanism’s rotation and an adhesive structure for the strain gauge, flexible hinges required a detailed design. According to the strain gauge sensing method, it was necessary to obtain the maximum stress at the minimum cutting thickness of the single-axis flexible hinge’s surface, so a single-axis parabolic flexible hinge was selected as shown in Figure 3; t is the minimum cutting thickness for the flexible hinge, l is the length of the parabolic section, h(x) is the cutting thickness for the flexible hinge, and b is the thickness of the flexible hinge section.

The cutting thickness for the flexible hinge could be expressed as

(1) $$h(x)=2a{\left(x-\frac{l}{2}\right)}^{2}+t,$$

The expression for the rotational stiffness of the single parabolic flexible hinge around the Z-axis is

(2) $$K_{\theta_{z}}=\frac{M_{z}}{\theta_{z}},$$

According to Castigliano’s second theorem,

(3) $$\theta_{z}=\frac{\partial U}{\partial M_{z}},$$

where U is the bending strain energy of a flexible hinge and can be expressed as

(4) $$U=\frac{1}{2}\int_{h}\frac{M_{z}^{2}}{EI_{z}(x)}dx,$$

where E is the elastic modulus and $I_{z}(x)=\frac{bh{\left(x\right)}^{3}}{12}$ is the moment of inertia around the Z-axis.

By combining Equations (2)–(4), we can obtain

(5) $$K_{\theta_{z}}=\frac{Eb}{12\int_{0}^{l}h{\left(x\right)}^{-3}dx},$$

Considering the stress concentration, the maximum stress of a flexible hinge can be expressed as

(6) $$\sigma_{\max}=k\frac{M_{z}}{W_{z}}=\frac{6kK_{\theta_{z}}\theta_{z}}{t^{2}b},$$

where $k=\frac{\varsigma +0.188}{\varsigma +0.014}$ is the stress concentration factor and ς=ρ/t; ρ is the curvature radius of the parabolic flexible hinge where the stress is highest.

On the basis of meeting the design objectives and ensuring that the maximum stress was less than the allowable stress, the parameters of the parabolic flexible hinge were determined as a=0.5, l=3.6 mm, t=1.25 mm, and b=3.45 mm. The maximum stress at the flexible hinge was 74.32 MPa.

**Figure 4:**
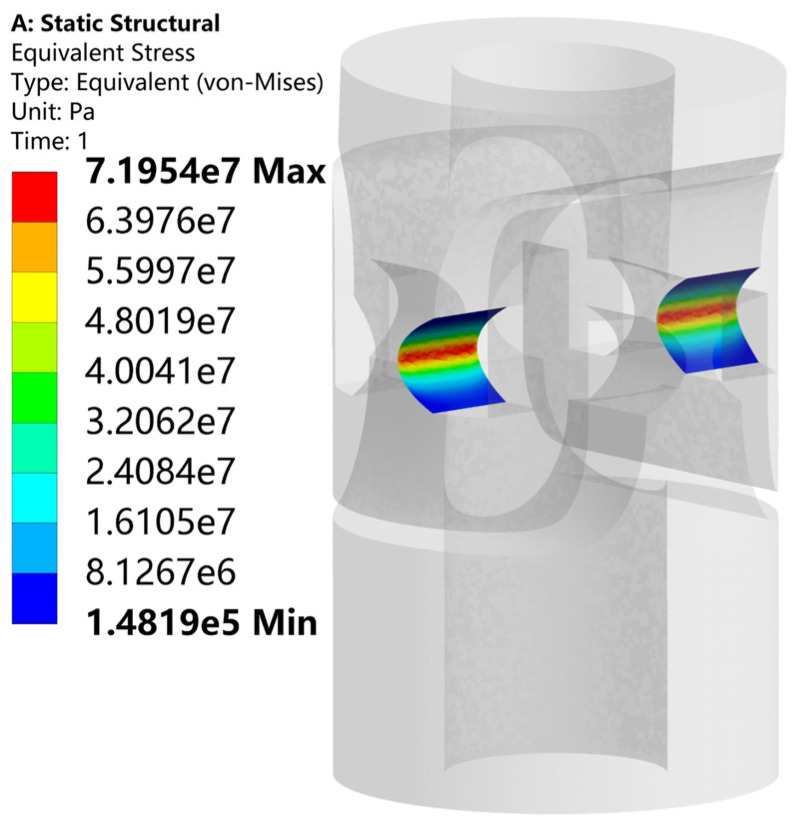
The stress of the flexible hinge.

According to the designed 1.5 mrad rotation angle, the stress of the flexible hinge was analyzed using ANSYS Workbench 2021 R1, as shown in Figure 4. It can be seen that the maximum stress at the flexible hinge was 71.95 MPa, which is consistent with the theoretical analysis and less than the yield strength of the material.

**Figure 5:**
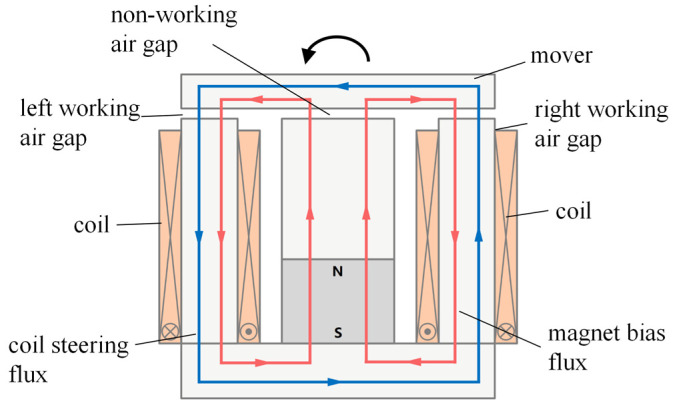
Diagrammatic representation of the HRAFSM’s operating principle.

The bias flux generated by the permanent magnet (red arrow in Figure 5) passes through the central yoke, the non-working air gap, the upper yoke/mover, the left and right working air gaps, and the left and right yoke, and finally returns to the permanent magnet through the lower yoke. When the upper yoke/mover is in a horizontal state, the magnet bias flux is evenly distributed in the left and right air gaps. Under no current, the forces on the left and right sides of the upper yoke are balanced, with no torque effect. The coil steering flux generated by the input current (blue arrow in Figure 5) passes through the left and right yokes, the upper yoke/mover, the left and right air gaps, and the lower yoke in a counterclockwise direction. The magnet bias and coil steering fluxes superimpose within the two working air gaps; in the left (right) working air gap; the two fluxes follow the same (opposite) directions (Figure 5), resulting in unequal magnetic induction intensities. This leads to unbalanced forces at the two ends of the upper yoke/mover, causing its rotation due to electromagnetic torque; the rotation direction changes with the direction of the current in the electromagnetic coil.

## 3. Dynamic Model

**Figure 6:**
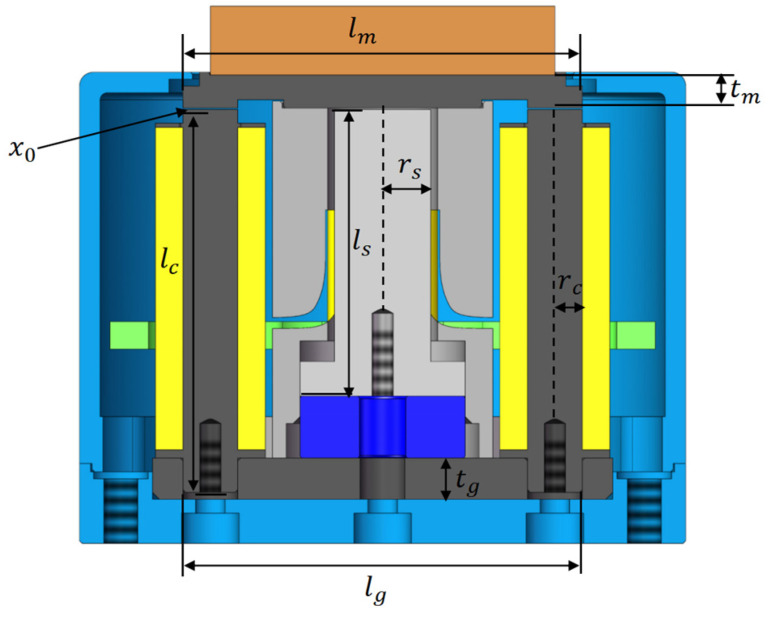
Component lengths within the HRAFSM structure.

Figure 6 shows the different components’ lengths in the HRAFSM structure, where $x_{0}$ is the initial working air gap thickness; $l_{m}$ is the equivalent length of the mover; $l_{s}$, $l_{c}$, and $l_{g}$ are the equivalent lengths of different parts of the yokes; $r_{s}$ and $r_{c}$ are the radii of the central and left/right yokes, respectively; and $t_{m}$ and $t_{g}$ are the thicknesses of the upper and lower yokes, respectively.

**Figure 7:**
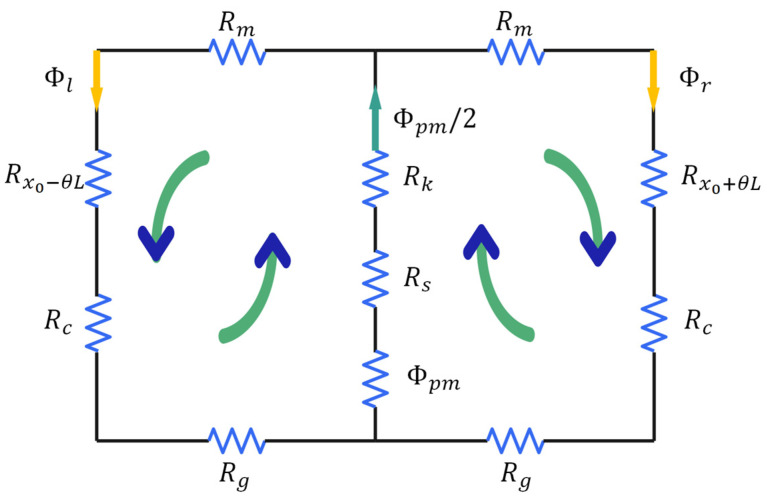
Equivalent model of the magnetic circuit generated by the permanent magnet.

The HRAFSM contains two types of magnetic circuits: one generated by the permanent magnet and the other by the current. The equivalent model of the magnetic circuit generated by the permanent magnet is shown in Figure 7, where $R_{pm}$ represents the reluctance of the permanent magnet; $R_{c}$ is the reluctance of the single yoke; $R_{m}$ and $R_{g}$ are half of the reluctance of the upper and lower yokes, respectively; $R_{x_{0}-\theta L}$ and $R_{x_{0}+\theta L}$ are the reluctance of the left and right working air gaps, respectively; $R_{k}$ is the reluctance of the non-working air gap; $\Phi_{pm}$ is the flux of the permanent magnet; and $\Phi_{l}$ and $\Phi_{r}$ represent the flux at the air gap of each branch.

When the mover rotates counterclockwise by an angle of θ, the thickness of the left/right working air gap changes by x=θL, where L is the radius of the rotation. According to Kirchhoff’s second law of magnetic circuits, the magnetic potential of the left and right magnetic circuits can be expressed as

(7) $$\Phi_{l}\left(R_{m}+R_{x_{0}-\theta L}+R_{c}+R_{g}\right)+\frac{\Phi_{pm}}{2}\left(R_{pm}+R_{s}+R_{k}\right)=0,$$

(8) $$\Phi_{r}\left(R_{m}+R_{x_{0}+\theta L}+R_{c}+R_{g}\right)+\frac{\Phi_{pm}}{2}\left(R_{pm}+R_{s}+R_{k}\right)=0,$$

where

(9) $$R_{x_{0}-\theta L}=\frac{x_{0}-x}{\mu_{0}S_{0}},R_{x_{0}+\theta L}=\frac{x_{0}+x}{\mu_{0}S_{0}},$$

where $\mu_{0}$ is the vacuum permeability, and $S_{0}$ is the cross-sectional area of the working air gap. Additionally, we know that

(10) $$\frac{\Phi_{pm}}{2}-\left(\Phi_{l}+\Phi_{r}\right)=0,$$

By combining Equations (7)–(10), we obtain

(11) $$\Phi_{l}=\frac{x_{0}+\theta L+\mu_{0}S_{0}\left(R_{m}+R_{c}+R_{g}\right)}{2x_{0}+2\mu_{0}S_{0}\left(R_{m}+R_{c}+R_{g}\right)}\frac{\Phi_{pm}}{2},$$

(12) $$\Phi_{r}=\frac{x_{0}-\theta L+\mu_{0}S_{0}\left(R_{m}+R_{c}+R_{g}\right)}{2x_{0}+2\mu_{0}S_{0}\left(R_{m}+R_{c}+R_{g}\right)}\frac{\Phi_{pm}}{2},$$

**Figure 8:**
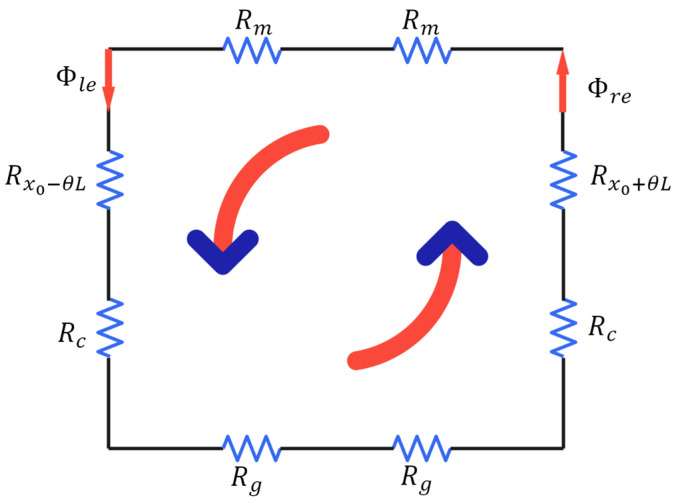
Equivalent model of the magnetic circuit generated by the current in the coil.

If an excitation current is applied to the left coil along the normal direction to the circuit with the current moving in (out) from the left (right), the coil generates a vertically downward magnetic field. The current then flows in from the right and out through the left in the right coil, generating an opposite magnetic field to the left side (Figure 8). 

According to Kirchhoff’s first law, the magnetic fluxes in the left and right magnetic circuits must be equal in magnitude. Moreover, according to Kirchhoff’s second law of magnetic circuits, the magnetic potential of the left and right magnetic circuits can be expressed as

(13) $$\Phi_{le}\left(R_{x_{0}-\theta L}+R_{m}+R_{c}+R_{g}\right)-\Phi_{re}\left(R_{x_{0}+\theta L}+R_{m}+R_{c}+R_{g}\right)=2NI,$$

where N is the number of turns in the left and right coils, and I is the current intensity applied to them. By combining Equations (13) and (14), we obtain

(14) $$\Phi_{le}=\Phi_{re}=\frac{\mu_{0}S_{0}}{x_{0}+\mu_{0}S_{0}\left(R_{m}+R_{c}+R_{g}\right)}NI,$$

The magnetic flux density at the left and right working gaps can then be obtained as

(15) $$B_{L}=\frac{\Phi_{l}+\Phi_{le}}{S_{0}}=\frac{\mu_{0}}{x_{0}+\mu_{0}S_{0}\left(R_{m}+R_{c}+R_{g}\right)}NI+\frac{x_{0}+\theta L+\mu_{0}S_{0}\left(R_{m}+R_{c}+R_{g}\right)}{2x_{0}+2\mu_{0}S_{0}\left(R_{m}+R_{c}+R_{g}\right)}\frac{\Phi_{pm}}{2S_{0}},$$

(16) $$B_{R}=\frac{\Phi_{r}-\Phi_{re}}{S_{0}}=\frac{\mu_{0}}{x_{0}+\mu_{0}S_{0}\left(R_{m}+R_{c}+R_{g}\right)}NI-\frac{x_{0}-\theta L+\mu_{0}S_{0}\left(R_{m}+R_{c}+R_{g}\right)}{2x_{0}+2\mu_{0}S_{0}\left(R_{m}+R_{c}+R_{g}\right)}\frac{\Phi_{pm}}{2S_{0}},$$

According to Maxwell’s stress tensor theory, the electromagnetic driving force at the left and right air gaps can be obtained, and then the electromagnetic torque $M_{m}$ applied to the mover can be expressed as

(17) $$M_{m}=K_{i}I+K_{m}\theta ,$$

where $K_{i}=\frac{NL\Phi_{pm}}{2x_{0}+2\mu_{0}S_{0}\left(R_{m}+R_{c}+R_{g}\right)}$ is the coupling coefficient between torque and current, and $K_{m}=\frac{\Phi_{pm}^{2}L^{3}}{8\mu_{0}S_{0}x_{0}+8\mu_{0}^{2}S_{0}^{2}\left(R_{m}+R_{c}+R_{g}\right)}$ is the stiffness caused by the magnetic circuit. It can be seen that the electromagnetic torque is not only related to the current but also to the rotation angle. As the rotation angle increases, the torque increases, exhibiting a negative stiffness effect.

**Figure 9:**
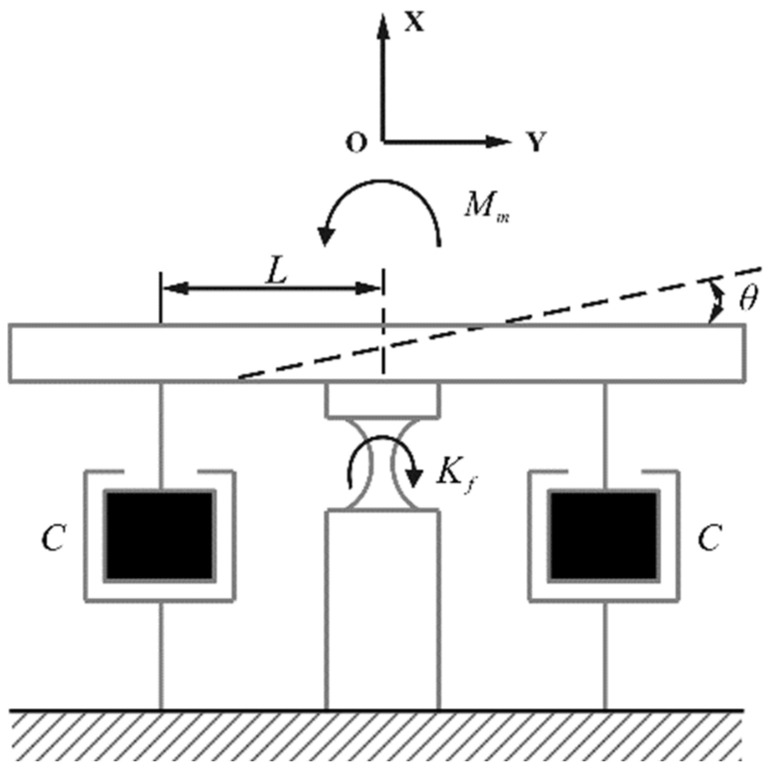
Equivalent mechanical model of the HRAFSM.

Figure 9 shows the equivalent mechanical model of the HRAFSM. According to the theory of vibration mechanics, the dynamic equation of the HRAFSM in the uniaxial direction can be expressed as

(18) $$J_{eq}\ddot{\theta }+2CL^{2}\dot{\theta }+K_{f}\theta =M_{m},$$

where $J_{eq}$ is the moment of inertia of the mover, C is the equivalent damping of the system, and $K_{f}$ is the rotational stiffness of the flexible support. Due to the negative stiffness effect generated by the magnetic circuit, the fundamental frequency obtained by the dynamic system will be lower than the natural frequency of the FSM structure.

By substituting Equation (17) into Equation (18) and performing the Laplace transform, a second-order transfer function between the rotation angle and the current is obtained as

(19) $$G(s)=\frac{\theta (s)}{I(s)}=\frac{K_{i}}{J_{eq}s^{2}+2CL^{2}s+K_{f}-K_{m}},$$

The parameters of G(s) can be obtained through system identification for the control algorithm design.

## 4. Controller Design

For the high-precision amplitude tracking control of the HRAFSM, we designed the PID + AIC controller. The basic principle of AICs is to use a controller to drive the target system [21]. Moreover, the controller transfer function must be the inverse of that of the target system for the controller output to be consistent with the target system’s input, achieving tracking of the target instruction. Filters play an important role in AICs, acting as independent blocks that take “error” as input and “expectation” as output. During this process, the error signal is used to adjust the filter parameters. We used (i) a linear least mean squares (LMS) adaptive filter to construct the AIC, and (ii) FIR filters to identify and describe the entire system, with a simple and convenient identification process and fewer adjustable weight coefficients. 

**Figure 10:**
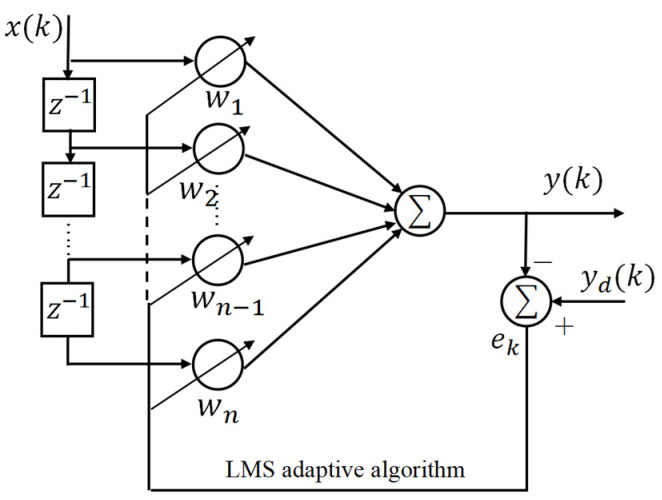
Diagram of a FIR filter based on the LMS adaptive algorithm.

Figure 10 shows the diagram of a FIR filter based on the LMS adaptive algorithm. The parameters of the adaptive filter are constantly changing during the adaptive process, causing the output signal y(k) to approach the desired signal $y_{d}(k)$.

The pulse transfer function of a FIR filter can be expressed as

(20) $$W(z)=w_{1}+w_{2}z^{-1}+w_{3}z^{-2}+\cdots +w_{n-1}z^{-N+1}+w_{n}z^{-N},$$

where $W_{k}^{T}=\left[\begin{array}{cccc} w_{1}(k) & w_{2}(k) & \cdots & w_{n}(k) \end{array}\right]$ is the weight vector of the FIR adaptive filter, and n is the length of the filter.

The output of the filter can be represented as

(21) $$y(k)=W_{k}^{T}X_{k}=\sum_{i=1}^{n}w_{i}(k)x(k-i+1),$$

where $X_{k}={\left[\begin{array}{cccc} x(k) & x(k-1) & \cdots & x(k-n+1) \end{array}\right]}^{T}$ is the input time series, and k is the number of iterations of the adaptive process.

The LMS algorithm is used to iterate the weight coefficients of FIR adaptive filters.

(22) $$W_{k+1}=W_{k}+\mu e_{k}X_{k},$$

where μ is a stable step size for the adaptive algorithm, and the error $e_{k}$ is

(23) $$e_{k}=y_{d}(k)-y(k)=y_{d}(k)-W_{k}^{T}X_{k},$$

**Figure 11:**
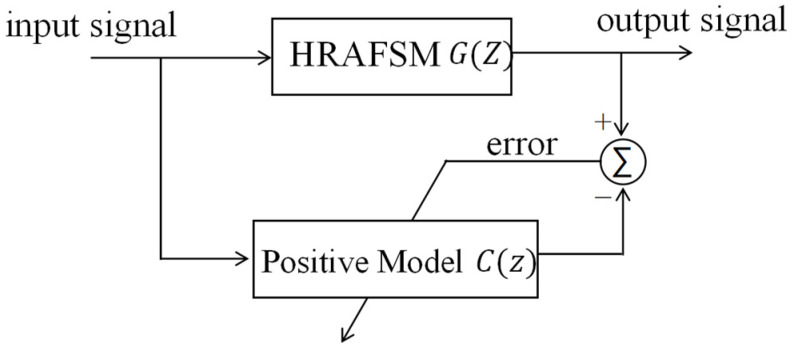
Block diagram of the positive model of the HRAFSM.

There are two types of adaptive inverse control, one is offline and the other is online [21], and the positive model and inverse model were determined to be offline in this research. The AIC construction process involved the building of a positive model based on the physical system (Figure 11) and an inverse model based on the positive model. In the positive model, the input signals were passed through an HRAFSM and a filter. The output angle of the HRAFSM was used as the expected signal, and the filter parameters were continuously adjusted on the basis of the difference signal between the filter output and the desired signal till the former became consistent with the real system’s output.

**Figure 12:**
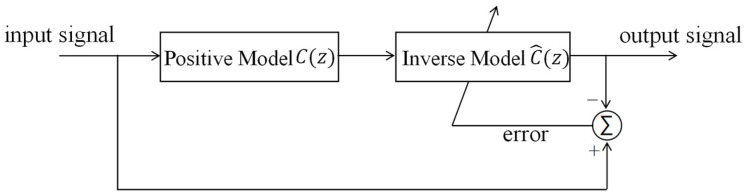
Block diagram of the inverse model of the HRAFSM.

Using the obtained desired positive model, we constructed an inverse model (Figure 12). The input signal was passed through the positive model and the filter successively. The filter parameters were continuously adjusted according to the error signal between the input signal and the filter output signal to make the two consistent.

**Figure 13:**
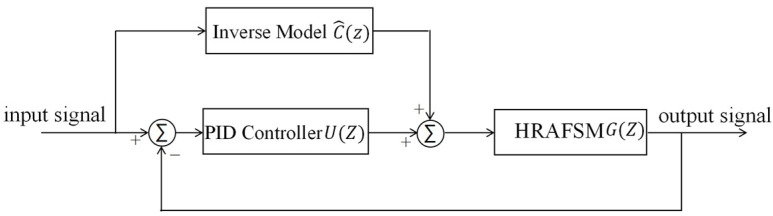
Schematic representation of the PID + AIC composite control system.

Theoretically, concatenating an AIC in a real system can achieve consistency between the input and output. However, the time delay in such systems can introduce errors in the process of constructing the AIC, resulting in a low control accuracy. Additionally, the difficulty in maintaining consistency between the mechanical and the strain-sensing zero positions leads to a constant output mechanical angle in the absence of input, which cannot be solved by the AIC. To improve control accuracy and eliminate steady-state mechanical angles, we designed a composite PID + AIC control (Figure 13), with both feedforward (AIC) and feedback (PID) loops.

According to the control diagram in Figure 13, the relationship between input and output signals can be obtained

(24) $$\frac{output}{input}=\frac{U(z)G(z)+\hat{C}(z)G(z)}{1+U(z)G(z)},$$

In order to verify the effectiveness of the controller through simulations, the transfer functions of the rotation angle around the x- and y-axes should be obtained by using the system identification method [22,23]. The time-domain response of the HRAFSM’s rotation angle around the x- and y-axes was obtained through frequency scanning. By using the system identification toolbox in Matlab/Simulink in combination with Equation (19), we obtained the transfer functions as

(25) $$G_{x}\left(s\right)=\frac{\theta_{x}\left(x\right)}{I_{x}\left(x\right)}=\frac{4.462\times 10^{7}}{s^{2}+352.2s+2.063\times 10^{7}},$$

(26) $$G_{y}\left(s\right)=\frac{\theta_{y}\left(s\right)}{I_{y}\left(s\right)}=\frac{5.079\times 10^{7}}{s^{2}+420.5s+2.086\times 10^{7}},$$

**Figure 14:**
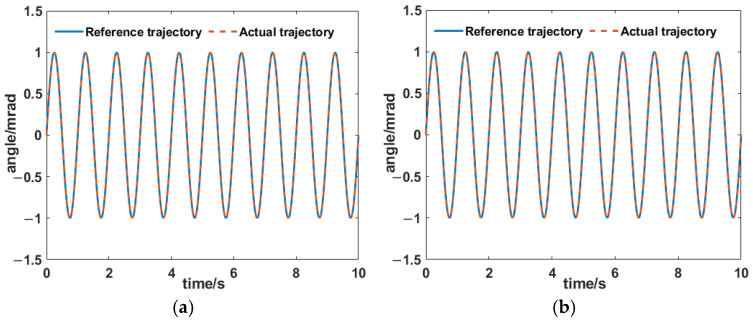
Tracking results for the PID + AIC composite control system at (**a**) 1 and (**b**) 10 Hz.

**Figure 15:**
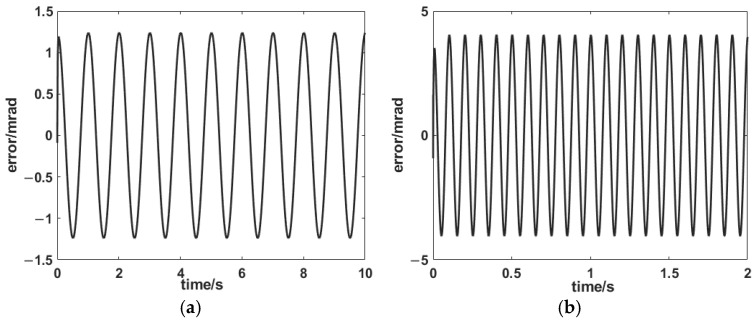
Tracking errors for the PID + AIC composite control system at (**a**) 1 and (**b**) 10 Hz.

As the two axes were decoupled, we used just the x-axis to validate the PID + AIC control strategy (Figure 14 and Figure 15). It can be seen that the control errors were 0.12% and 0.4% at 1 Hz and 10 Hz, respectively. It demonstrated excellent target tracking.

## 5. Experiment

**Figure 16:**
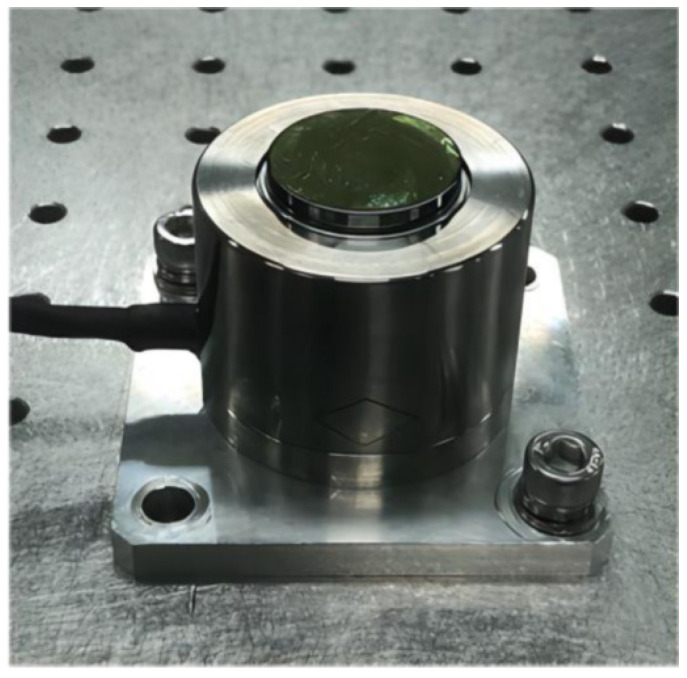
Proposed HRAFSM prototype.

Figure 16 shows the prototype of the designed HRAFSM. In order to ensure high precision and minimize assembly errors, the flexible hinge adopts an integrated processing method and uses a TC4 titanium alloy as the material. The dimension parameters are displayed in Table 1.

**Figure 17:**
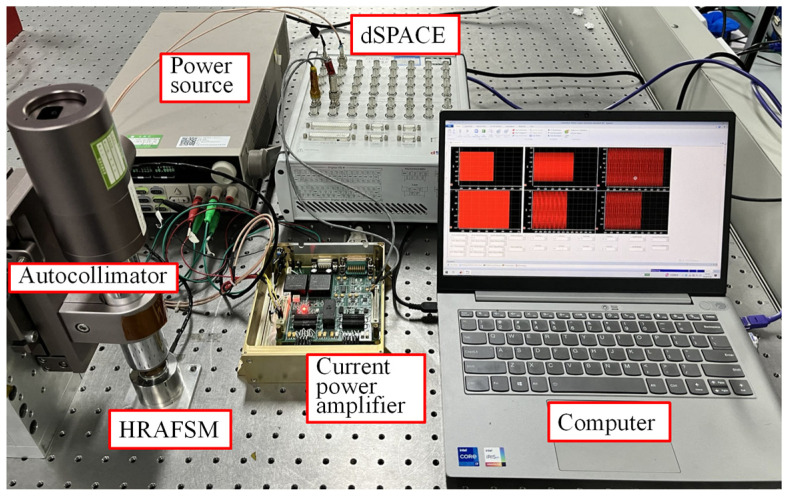
Experimental test setup for the proposed HRAFSM.

The HRAFSM experimental system included a computer, dSPACE, a current power amplifier, the HRAFSM, and an autocollimator (Figure 17). The HRAFSM employed strain sensing, which cannot directly provide the angle of rotation, and hence was calibrated using an autocollimator. The control algorithm was designed using Matlab/Simulink R2022b, and compiled and tested using the dSPACE ControlDesk 2020 for the control effects. Simultaneously, dSPACE achieved strain signal acquisition and voltage signal transmission. The self-developed current drive mainly converted voltage signals into current signals and subsequently drove the HRAFSM.

### 5.1. Performance Test

**Figure 18:**
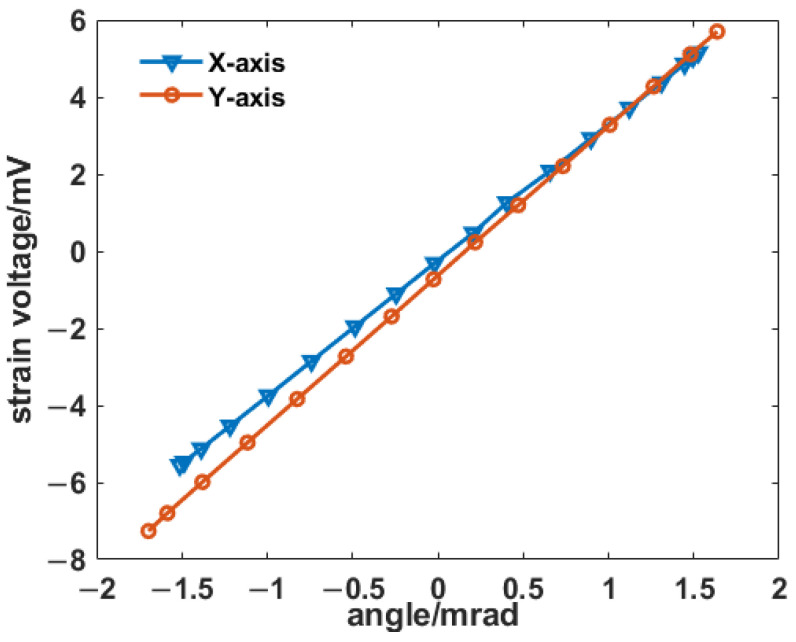
Relationship between strain voltage and rotation angle.

The proposed HRAFSM uses the strain sensors and first calibrates the relationship between the strain voltage and the rotation angle through the autocollimator within the total travel range, as shown in Figure 18. Testing results revealed a good linear relationship between the rotation angle of the two axes and their corresponding strain voltages. Therefore, this sensing method could be applied to the closed-loop feedback control of the HRAFSM. The sensitivities of the x- and y-axes were 3.526 mV/mrad and 3.886 mV/mrad, respectively, with both axes achieving an expected rotation angle of ±1.5 mrad.

**Figure 19:**
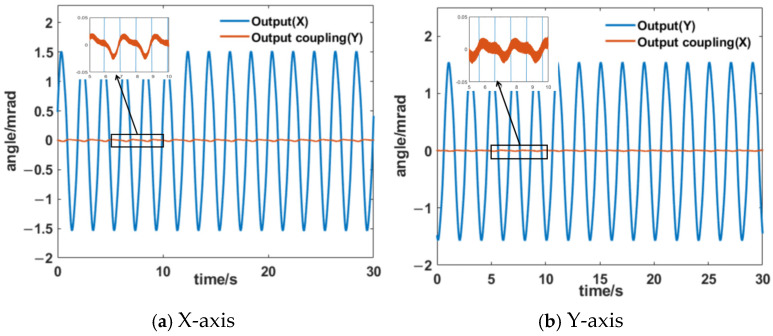
Decoupling tests for the x- and y-axes of the HRAFSM.

The two axes of the HRAFSM were structurally decoupled through flexible hinges. To evaluate the decoupling effect, sinusoidal excitation signals were applied to both axes separately and the coupling response was recorded (Figure 19). Under a sinusoidal rotation of 1.5 mrad, the coupling magnitudes of the two axes of the designed HRAFSM were 0.02 mrad and 0.015 mrad, respectively, with coupling degrees of approximately 1.3% and 1%. It indicated good decoupling characteristics.

**Figure 20:**
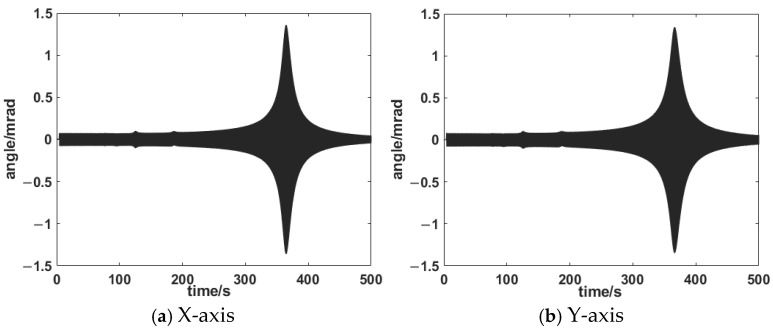
Time-domain response diagram.

**Figure 21:**
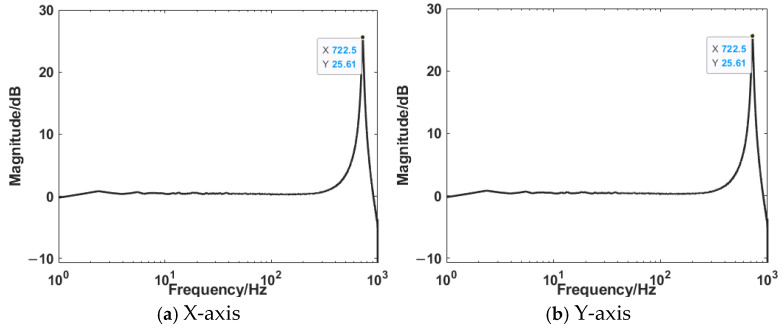
Frequency-domain response diagram.

In order to obtain the dynamic characteristics of the HRAFSM, a sine sweep signal was used to sweep the mechanism, with a fixed amplitude of 0.1 V and a frequency range of 0.1 Hz to 1 kHz. The time-domain response and frequency-domain response are shown in Figure 20 and Figure 21. From the figure, it can be seen that the first-order natural frequencies of the x-axis and y-axis are 722.5 Hz and 724.7 Hz, respectively.

### 5.2. Tracking Test

**Figure 22:**
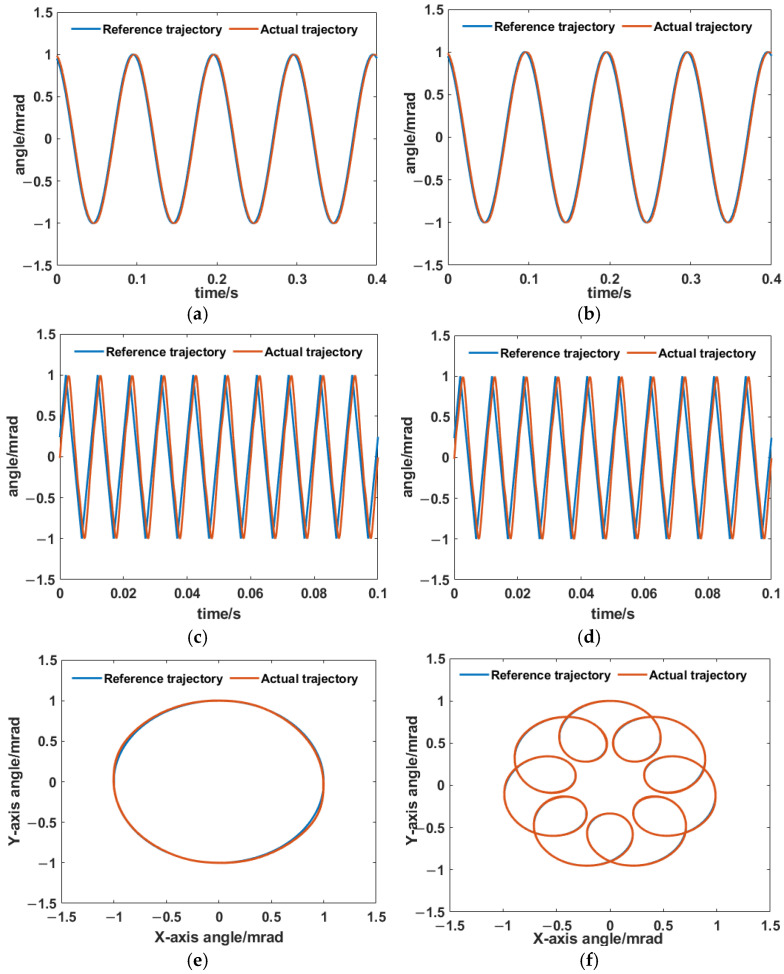
The HRAFSM’s tracking control for (**a**,**b**) the x- and y-axis sinusoidal trajectories, respectively; (**c**,**d**) x- and y-axis triangular trajectories, respectively; (**e**) a circular trajectory; and (**f**) a complex trajectory.

To examine the tracking ability of the HRAFSM under the PID + AIC controller, we first conducted a single-axis sinusoidal trajectory tracking test, as shown in Figure 22a,b. The amplitude tracking errors of the x- and y-axes were 0.1% and 0.14%, respectively. A 100 Hz triangular trajectory was used to test the closed-loop control performance, as shown in Figure 22c,d. It demonstrated excellent tracking performance. We further investigated the 2-DoF trajectory tracking performance of the HRAFSM using a simple circular reference trajectory with a frequency of 10 Hz and a complex reference trajectory defined in [24], which were chosen the reference trajectories. The experimental results are shown in Figure 22e,f; it can be seen that the HRAFSM can track complex trajectories with high accuracy under the designed PID + AIC control method.

## 6. Conclusions

This study presents the design of an HRAFSM with integrated strain sensing and a combined PID +AIC control algorithm. The HRAFSM is guided by flexible support to achieve kinematic decoupling. The theoretical analysis revealed a second-order transfer function between the rotation angle and the current for this system. The experimental results indicate that the use of strain sensors in the HRAFSM can help achieve a compact design. Additionally, the composite PID + AIC control method achieves high-precision tracking trajectory control for the HRAFSM. The designed HRAFSM in this study has a relatively small travel range; we will design an HRAFSM with a larger rotation angle.

## Figures and Tables

**Table 1:** Parameters of the HRAFSM.

Parameters	Value
Mass	148 g
External dimensions	Φ44 × 39 mm^3^
Mirror dimensions	Φ25 × 5 mm^3^
$l_{m}$	12.5 mm
$l_{c}$	28.8 mm
$l_{g}$	12.5 mm
$t_{m}$	1.8 mm
$t_{g}$	2.5 mm
$r_{c}$	2.0 mm
$x_{0}$	0.2 mm

## Data Availability

Data are contained within the article.

## Abbreviations

The following abbreviations are used in this manuscript:

PID	Proportional-integral-derivative
AIC	Adaptive inverse control
HRAFSM	Hybrid reluctance-actuated fast steering mirror
FSM	Fast steering mirror
DoF	Two degrees of freedom
